# Brugada Phenotype Following a Cocaine Overdose

**DOI:** 10.7759/cureus.63861

**Published:** 2024-07-04

**Authors:** Ammar F Chauhdri, Patrick Bruss, Alvin Tran

**Affiliations:** 1 Emergency Medicine, ProMedica Monroe Regional Hospital, Monroe, USA; 2 Medicine, University of Michigan, Ann Arbor, USA

**Keywords:** connexin 43, cardiotoxicity, sodium channels, electrolyte abnormality, cocaine, ecg, brugada type 1, brugada syndrome

## Abstract

Brugada syndrome is a rare cardiac condition characterized by distinctive electrocardiogram patterns, predisposing individuals to fatal arrhythmias. While primarily linked to a loss-of-function mutation in the SCN5A gene, acquired forms of the syndrome have been associated with various factors, including drug use. We present a case of a 31-year-old female who presented to the emergency department unresponsive following cocaine use and developed type 1 Brugada ECG patterns alongside an incomplete right bundle branch block in V1-V3, ST elevations with biphasic waves, and diffuse repolarization abnormalities with J point deviations while in the intensive care unit. This study aimed to discuss the complexity of managing drug-induced Brugada-like findings and highlights the need for further research into the mechanisms underlying cocaine-induced cardiac effects. We aimed to discuss potential mechanisms for the impact of cocaine as its role as a sodium channel blocker and its potential effects on connexin 43 in the context of Brugada syndrome. This study also reinforced the importance of differentiating between true Brugada syndrome and other similar ECG changes for appropriate care management.

## Introduction

Brugada syndrome is a rare condition linked to fatal cardiac arrhythmias and it is characterized by distinct electrocardiogram (ECG) patterns, including right bundle branch block and ST-segment elevations in specific leads [1]. The three main ECG patterns notable for Brugada syndrome are as follows: type 1, characterized by coved ST-segment elevation followed by negative T waves; type 2, presenting a saddleback appearance of T waves with ST-segment elevation or J point elevation followed by positive or biphasic T waves; and type 3, showing either coved or saddleback appearance with ST-segment elevation. Clinical criteria include a family history of sudden cardiac death, ventricular arrhythmias, and symptoms like syncope [2]. A combination of type 1 Brugada pattern and at least one clinical criterion confirms the syndrome, while type 2 or type 3 patterns with clinical criteria suggest it [2].

Brugada syndrome is thought to have a genetic component and is primarily associated with a loss-of-function mutation in the SCN5A gene, although mutations in other genes have also been implicated. Brugada syndrome follows an autosomal dominant inheritance pattern but can demonstrate variable expressivity and reduced penetrance [1]. However, only a minority of clinically diagnosed cases show this genetic link. Experimental studies propose that alterations in cardiac ion currents (particularly an increase in transient outward current) may contribute to the syndrome's development [3]. Interventions or conditions that modulate these currents, such as medications, electrolyte imbalances, and illicit drug use (such as cocaine) [1], can mimic the ECG pattern seen in Brugada syndrome, suggesting the potential for acquired forms of the condition [3]. The pathophysiology remains somewhat uncertain, but it is thought these abnormalities in cardiac ion channels are what eventually lead to ventricular arrhythmias. Management for individuals showing symptoms typically involves implantable cardioverter-defibrillator (ICD) placement, pharmacological therapy like quinidine, or radiofrequency ablation. However, the approach to asymptomatic individuals with ECG abnormalities requires careful risk assessment and personalized management strategies [1,2].

Cocaine has been linked with Brugada syndrome in a few case studies to date. In a previous case report following the prognosis of a 32-year-old male with a history of cocaine and nicotine abuse presenting with a Brugada ECG pattern unmasked by cocaine use, the authors propose that cocaine's sympathomimetic effects and sodium channel blocking properties can induce transient Brugada-like patterns in individuals without a genetic predisposition to Brugada syndrome [4]. These patterns, observed after cocaine use, highlight the importance of distinguishing drug-induced ECG changes from genuine Brugada syndrome to ensure appropriate management. While treatments for cocaine-related cardiac complications exist, the long-term consequences and outcomes of drug-induced Brugada-like patterns remain uncertain [4].

In this study, we aimed to present the unique ECG findings that are consistent with those of type 1 Brugada in a 31-year-old female who was brought to the ED unresponsive with positive lab findings for cocaine, fentanyl, benzodiazepines, and tetrahydrocannabinol (THC). On initial evaluation, the patient was severely acidotic and had findings of bidirectional ventricular tachycardia which was resolved with electrolyte replacement and treatment. However, after being admitted to the ICU, the patient developed Brugada syndrome ECG patterns approximately 9 h after the resolution of her bidirectional ventricular tachycardia. We aimed to use this case to investigate potential mechanisms relating cocaine use with the induction of Brugada syndrome, as well as its implications for treatment.

## Case presentation

**Figure 1:**
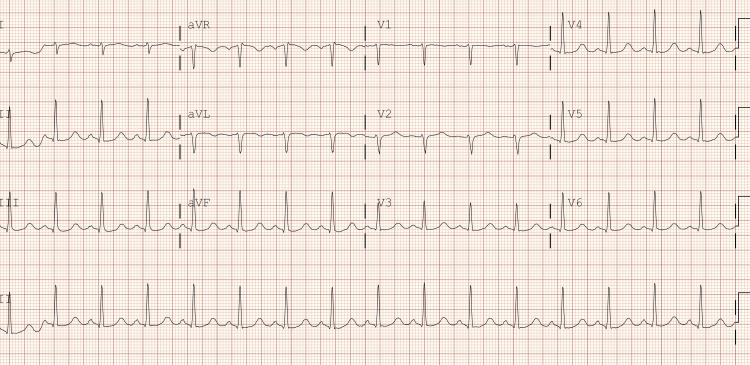
ECG obtained in the ED after successful resuscitation (right axis deviation, QTc 521 ms).

**Figure 2:**
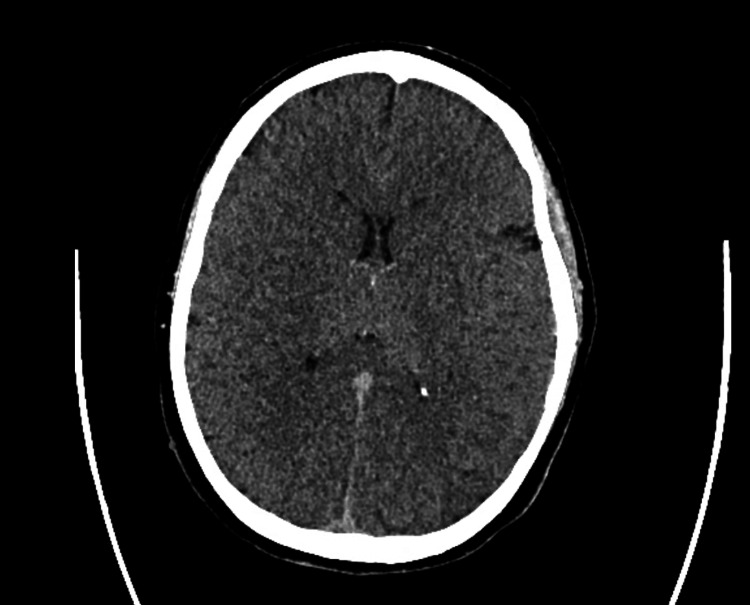
Reduced gray-white matter differentiation throughout the brain with partial effacement of the ventricular system concerning diffuse cerebral edema in the setting of hypoxic-ischemic encephalopathy.

The patient was a 31-year-old female who arrived at the emergency department after being found unresponsive following an episode where she and a friend lost consciousness after using an unknown substance. The family denies any known hereditary diseases. Upon arrival, she required immediate intubation and cardiopulmonary resuscitation (CPR). Her initial ECG findings after the return of spontaneous circulation were consistent with bidirectional ventricular tachycardia, right axis deviation, terminal R wave, elongated QT interval, and a wide QRS complex. Resuscitative efforts included CPR, epinephrine administration, IV calcium gluconate, IV magnesium sulfate, and fluid resuscitation with lactated ringers and vasopressors. Laboratory findings revealed severe acidosis, leukocytosis, elevated ammonia, lactate, glucose, and abnormal liver enzyme levels, along with a positive urine drug screen for benzodiazepines, THC, cocaine, and fentanyl. Following successful resuscitation, the patient’s physical examination findings included fixed and dilated pupils, irregular rhythm with diminished carotid pulses, respiratory distress, and unresponsiveness. A subsequent ECG performed in the emergency room showed resolution of the ventricular tachycardia but revealed a prolonged QTc interval of 521 ms and right axis deviation (Figure 1). CT imaging revealed cerebral edema, and the patient was transferred to the ICU for further management, including intravenous fluid resuscitation, antibiotic therapy, sedation, and vasopressor support (Figure 2).

**Figure 3:**
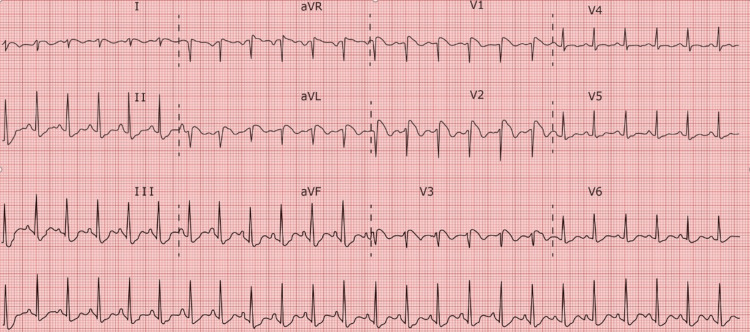
Sinus tachycardia - an incomplete right bundle branch block in V1-V3 with ST elevations and biphasic waves. The image also shows diffuse repolarization abnormalities with J point deviations throughout various leads, suggestive of a type 1 Brugada pattern.

The patient was admitted to the ICU where an arterial line was inserted to enable continuous hemodynamic monitoring, while a neurology consultation prompted the initiation of continuous EEG monitoring to investigate potential subclinical seizures. Throughout the afternoon, the patient displayed persistent tachycardia, leading to the administration of Lopressor to manage heart rate. An ECG was also collected 12 h after the first ECG and was suggestive of a type 1 Brugada pattern (Figure 3).

**Figure 4:**
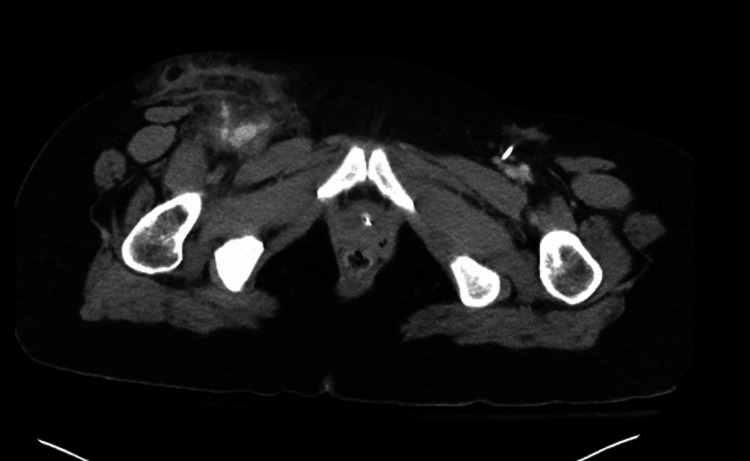
Irregularity within the right groin with possible pseudoaneurysm or hematoma with extravasation.

To address the cerebral edema identified on the CT brain scan, the patient received 3% hypertonic saline at a rate of 30 cc/h, alongside escalated pressor support. A CT abdominal imaging also showed an irregularity within the right groin, possibly indicating a pseudoaneurysm or hematoma (Figure 4). Despite these interventions, the significant cerebral edema and hypoxic injury prompted a consultation with neurosurgery, who deemed invasive interventions unlikely to improve the prognosis. Consequently, palliative care discussions ensued, culminating in a mutual decision with the family to transition the patient to a do-not-resuscitate (DNR) status. Sedation was discontinued on the second day of admission, leading to a brain death evaluation revealing no evidence of brain activity in the early afternoon. A repeat evaluation was scheduled for the following day, during which sedation remained off, and no clinical changes were observed. Another brain death evaluation, including an apnea challenge, confirmed the absence of brain activity on the third day. With the family present, mechanical and chemical support was withdrawn, with the patient entering asystole shortly thereafter, and the time of death was recorded on day three of admission, approximately 79 hours after arrival to the ED. The final diagnoses encompassed multiple contributing factors, including cardiac arrest secondary to drug overdose, accidental drug overdose, cocaine use disorder, acute respiratory failure with hypoxia and hypercapnia, anoxic brain injury, transaminitis, metabolic acidosis, acute kidney injury, and cerebral edema secondary to anoxia.

## Discussion

Cocaine has been linked to a multitude of acute cardiovascular findings and is very well studied in regard to its physiological cardiac effects. However, given the rarity of Brugada-like ECG findings in general (and even more so with cocaine use as a probable trigger), the pathophysiology for exactly how cocaine may lead to Brugada-like patterns is not known at this time. We aimed to use this study to propose potential mechanisms by which the use of cocaine may induce these findings, as well as suggest potential treatment pathways to be explored further.

The first mechanism in which cocaine may lead to Brugada-like ECG findings is through its sodium channel blocking. In Brugada syndrome, findings associated with a loss-of-function mutation in the SCN5A gene reveal alterations in the encoding of alpha subunits of myocardial sodium channels. These genetic defects result in defective sodium channels that decrease sodium influx while enhancing outward current, thereby leading to a plateaued action potential and increasing susceptibility to reentry ventricular arrhythmias [4,5]. Cocaine is a known cardiac sodium channel blocker and inhibits these channels by preferentially binding to open or inactivated states, inducing concentration-dependent inhibition and slowing channel repriming, suggesting a pore-blocking mechanism and stabilization of the channels in an inactivated state [6]. Therefore, we predict that in a mechanism similar to that of the loss of function mutation within the SCN5A gene, cocaine may either directly produce Brugada-like ECG findings or may actually serve to unmask underlying Brugada syndrome in otherwise asymptomatic patients through the decreased cardiac sodium influx [4].

Another potential mechanism may involve cocaine’s effect on cardiac connexin 43 gap junction proteins. In Brugada syndrome, alterations in connexin 43 (Cx-43) can disrupt intercellular communication in the heart, potentially contributing to arrhythmogenesis [7]. Studies investigating the effects of cocaine on cardiac Cx-43 have shown increased dephosphorylation of Cx-43 after cocaine exposure in rat models, which correlates negatively with the distribution of contraction band necrosis (CBN), a common myocardial abnormality associated with cocaine abuse [8]. These changes in Cx-43 profiles, particularly the increased localization of dephosphorylated Cx-43 in regions adjacent to CBN, may disrupt the normal propagation of electrical signals in the heart [7]. Since Brugada syndrome manifests as ventricular arrhythmias and is frequently associated with ECG patterns resembling right bundle branch block, alterations in connexin function could significantly contribute to the arrhythmogenic setting observed in this syndrome and contribute to the ventricular arrhythmias found [9].

Additionally, there is evidence that the permeability of Cx-43 gap junctions is regulated by the cell’s intracellular calcium concentration [10]. Cocaine is also known to block both calcium and sodium channels, further creating electrolyte abnormalities conducive to disrupting intercellular communication controlled by Cx-43 [11]. These disrupted intercellular communications mediated by Cx-43 alterations could thereby predispose individuals to the characteristic ventricular arrhythmias observed in Brugada syndrome. Furthermore, since Brugada syndrome is often associated with ST-segment elevation in the right precordial leads of the ECG, these cocaine-induced changes in Cx-43 could contribute to the development of the characteristic ECG patterns seen in acquired Brugada syndrome [12]. Therefore, the disruption of Cx-43-mediated intercellular communication by cocaine could be a contributing factor to the development of Brugada syndrome in patients with a history of cocaine abuse.

Studies also suggest that there is an observed decrease in Cx-43 expression in the myocardium of individuals with Brugada syndrome compared to control myocardium, suggesting a potential role for altered intercalated disc composition in the pathogenesis of Brugada syndrome [7]. Given the aforementioned effects of cocaine on Cx-43 and that Cx-43 is crucial for facilitating electrical coupling between cardiomyocytes, diminished expression of this protein may lead to impaired intercellular communication and electrical uncoupling among cardiomyocytes. Furthermore, the correlation between reduced Cx-43 expression in the myocardium and the Brugada syndrome phenotype observed in the SCN5A-knockout mouse model demonstrates a clear correlation with the human phenotype [7,13]. This disruption in electrical connectivity could contribute to the arrhythmogenic substrate observed in Brugada syndrome. Further research into the specific mechanisms linking Cx-43 alterations to the pathogenesis of Brugada syndrome in the context of cocaine exposure is warranted to fully elucidate this relationship and identify potential therapeutic targets.

Also of note is that the patient was severely acidotic on arrival to the ED with an initial venous blood gas pH of 6.694. For those with Brugada syndrome, severe acidosis may cause the sympathetic nervous system to become activated as a compensatory response [14], intended to stabilize the internal environment of the body. This can then affect cardiac electrophysiology by greatly impacting cardiac rhythm and perhaps raising the chance of arrhythmias [15]. The myocardium may become even more unstable due to the disrupted autonomic balance, increasing its susceptibility to the usual arrhythmic episodes associated with Brugada syndrome [14]. Autonomic tone is known to affect Brugada syndrome, therefore these alterations can affect those who have it more severely [14]. Usually, sympathetic activity reduces the symptoms of the condition while vagal dominance worsens them [16]. Hence, depending on the individual’s physiological reaction and the severity of the acidosis, the sympathetic response caused by the illness may either mitigate or conceal distinctive signs of Brugada syndrome [17]. One could argue that for the patient in this case the ECG abnormalities were due to the acidosis. We doubt this is the case due to the fact that the acidosis was improving. A repeat arterial blood gas (ABG) in the ICU 2 h prior to the ECG in Figure 3 was obtained and showed a pH of 7.27. While still not a normal value, it is significantly improved from the initial 6.694. In addition, the ECG in Figure 1 was obtained while the patient was acidotic and this ECG does not show the Brugada pattern.

This patient also had co-ingestion of other substances specifically benzodiazepines THC and fentanyl. There are reports of Brugada syndrome associated with an overdose of both THC and fentanyl. However, these reports are from patients who usually have polysubstance issues and have multiple drugs in their system at the time of evaluation. In the present case, we can not definitively say that cocaine was the sole cause of all of these abnormalities. We do feel that cocaine is the most likely culprit due to its effects on the sodium channels of the myocardium. The ECG abnormalities associated with Brugada syndrome are secondary to the genetic abnormalities of the sodium channels of the myocardium. For this case, we postulate that the patient has acquired the ECG abnormalities secondary to the ingestion of cocaine. So instead of being born with abnormal sodium channels of the myocardium, this patient acquired them by ingesting significant amounts of cocaine which caused an acute pathology of the sodium channels mimicking Brugada syndrome. While it is true that the patient may have had undiagnosed Brugada syndrome, which was revealed by ingestion of the substances, we find that less likely based on her history or lack thereof.

It is important to approach cases of cocaine-induced changes resembling Brugada syndrome with caution, distinguishing between actual Brugada syndrome and Brugada-like findings. While cocaine-induced alterations in cardiac physiology may mimic the characteristic ECG patterns of Brugada syndrome, the underlying mechanisms and long-term implications might differ, and further research is needed to determine the precise relationship between cocaine exposure and the development of Brugada-like findings in order to clarify whether these changes represent a true manifestation of acquired Brugada syndrome or a distinct cardiotoxic effect of cocaine. In the present case, there had been no previous extensive genetic or cardiological workups, making it difficult to determine whether or not she was genetically predisposed to Brugada syndrome. However, given that the patient did have a type 1 Brugada ECG finding in addition to experiencing ventricular arrhythmias on arrival to the ED, we believe that she did meet the criteria to be diagnosed with Brugada syndrome and that the cardiotoxic effects of her cocaine use may have contributed to unveiling it.

Comprehensive investigations are needed to understand the potential reversibility of these cocaine-induced alterations and their clinical significance. At this time, cocaine has no approved antidote in case of overdoses, so patients with cocaine-related Brugada-like ECG patterns should be treated with standard supportive therapy, which may include nitroglycerin, benzodiazepines, and electrolyte management [4]. Active cooling and sodium bicarbonate infusions have also been found to help normalize blood pH in cocaine overdose and help reverse cardiac conduction issues [18]. Isoproterenol, often used alongside quinidine, has also demonstrated effectiveness in restoring normal ST segment elevation in individuals with Brugada syndrome and in helping to manage electrical storms, especially in pediatric cases [19]. Following acute care, patients should follow-up to discuss more permanent solutions as needed, such as ICD placement, pharmacological therapy like quinidine, or radiofrequency ablation as warranted. Given the potential implications for patient management and treatment strategies, further research efforts are needed to determine the pathophysiological mechanisms underlying cocaine-induced cardiac effects and their association with Brugada-like findings.

## Conclusions

While cocaine-induced changes in heart function can resemble the ECG patterns seen in Brugada syndrome, it's essential to differentiate between true Brugada syndrome and similar findings caused by cocaine. Although there are indications that cocaine might contribute to Brugada syndrome by affecting cardiac sodium channels and connexin 43, further investigation is necessary to fully grasp these mechanisms and their link to Brugada-like findings in individuals with a history of cocaine abuse. The case discussed highlights the challenges in diagnosing and managing such scenarios, especially when substance abuse is involved without prior genetic or cardiac evaluations. While this patient's clinical presentation is indicative of Brugada syndrome, further exploration is required to determine if her condition was induced by cocaine or simply unmasked by it. In conclusion, extensive research is necessary in order to better understand the mechanisms by which cocaine may lead to or unmask Brugada syndrome and to develop more effective treatment strategies for individuals affected by this complex interaction.
